# Supplementary material for: Psychometric Properties of Interpersonal Regulation Questionnaire for Chinese College Students: Gender Differences and Implications for Well-Being
Source: Behav Sci (Basel). 2023 Jun 16;13(6):507. doi: 10.3390/bs13060507 (PMC10295745; doi:10.3390/bs13060507)
Supplement: Supplementary file 1 [file behavsci-13-00507-s001.zip › behavsci-2422418-supplementary.pdf]

Table S1. Estimation of latent gender differences across four IRQ factors.

	Male (n=209)	Female (n=346)	SE	*p*	*Cohen's d*
Negative Tendency	0	.364	.100	0.000	.304
Negative Efficacy	0	.474	.091	0.000	.376
Positive Tendency	0	.032	.100	0.748	.031
Positive Efficacy	0	.002	.097	0.987	.002

Table S2. Standardized factor loadings for the confirmatory factor analysis models.

Model	χ^2	*df*	CFI	RMSEA[90% CI]	SRMR
Model with four-factor	366.77	100	.890	.070[.063-.078]	.088
Model with one higher-order factor	432.46	100	.864	.079[.071-.086]	.075

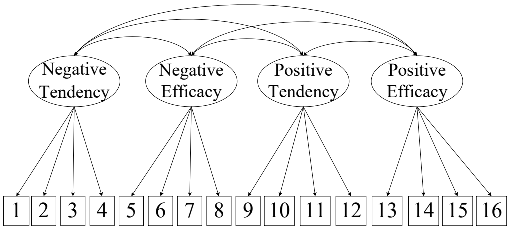

The four-factor model

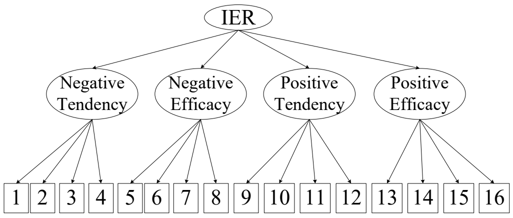

Model with one higher-order factor

Figure S1. Graphical representation of the confirmatory factor models. *Note.* IER = interpersonal emotion regulation.
